# ‘The men who made the breakthrough’: How the British press represented Patrick Steptoe and Robert Edwards in 1978

**DOI:** 10.1016/j.rbms.2017.07.002

**Published:** 2017-08-16

**Authors:** Katharine Dow

**Affiliations:** University of Cambridge, Free School Lane, Cambridge CB2 3RQ, UK

**Keywords:** IVF, media, Patrick Steptoe, Robert Edwards, Louise Brown, 1978

## Abstract

This article examines how the British press represented Patrick Steptoe and Robert Edwards in the story of the birth of the first ‘test-tube baby’, Louise Brown. In 1978, the British press represented the birth of Louise Brown as both a success and a source of hope. The main pairs of protagonists in this story were Steptoe and Edwards and Lesley and John Brown, who metonymically represented British science and infertile couples, respectively. In the dominant ‘success’ narrative of the birth of Louise Brown as depicted in the British press in 1978, Edwards and Steptoe seemed to embody ‘British’ values of industriousness, perseverance, altruism, ingenuity and teamwork. Thus, their success was simultaneously a British success. With Louise Brown’s birth, in-vitro fertilization came to stand for the potential happiness of infertile people and a bright future for British science and industry.

## Introduction

Scholars and journalists alike typically cite the birth of Louise Brown, the world’s first ‘test-tube baby’, in Oldham, Greater Manchester, UK on 25 July 1978 as the origin story of in-vitro fertilization (IVF). The birth was not only a medical ‘breakthrough’, but also a media sensation. Important scholarly and journalistic work has been done on the international (and particularly American-led) media response to this key event in 20th-century medical science (Condit, 1994, Harris, 2006, Henig, 2004, Nelkin and Raymond, 1980, Seguin, 2001, Van Dyck [van Dijck], 1998), and on how it developed existing tropes about the artificial creation of life in the English-speaking world (Squier, 1994, Turney, 1998). Social scientists have also shed light on the different national responses to subsequent IVF firsts, helpfully drawing attention to the way that this technology interacts with specific social contexts and media histories (Bharadwaj, 2000, Birenbaum-Carmeli et al., 2000, Shalev and Lemish, 2012; see also Michelle, 2007).

Although many scholars of IVF are aware of media representations of this technology, research tends to focus on individual patients’ experiences. In particular, very little work has considered seriously the role of the British media in the history of IVF. Yet, as Sarah Franklin has written, ‘These representations are an important public source of both formal knowledge and commonsense understandings of the experience of infertility and the rapidly expanding field of “test-tube baby” science’ (1990: 201). They also help shape policy towards IVF and the other forms of research and technology that have sprung from it (Franklin, 2013, Johnson et al., 2010, Mulkay, 1997, Petersen, 2001, Williams et al., 2003).

In 1978, there was public disquiet across the world, and particularly in the USA, about the birth of the world’s first IVF baby, and concern about what it might mean for technology and for humans’ relationships with nature, god and each other (Harris, 2006, Henig, 2004, Nelkin, 1987, Nelkin and Raymond, 1980). The British researchers who had brought about Louise Brown’s in-vitro conception, consultant gynaecologist Patrick Steptoe and research scientist Robert Edwards, had been criticized for their experimental work on IVF in the late 1960s and early 1970s (Edwards and Steptoe, 1980), and embryological research was to face intense scrutiny in the 1980s (Mulkay, 1997). However, in 1978, the British press’ story of IVF was an overwhelmingly positive tale of happiness for Louise’s parents, Lesley and John Brown, and success for Edwards and Steptoe, which represented hope for other infertile couples and demonstrated the country’s excellence in medical research and innovation.^1^ In the UK, although some newspapers did report that the technique was controversial, they rarely, if ever, substantiated these claims with any specific examples.^2^ Across broadsheets and tabloids, the dominant narrative was that Louise’s birth was good news for both her family and the country. The celebration of ‘firsts’ is one clear point of intersection between scientific and journalistic thinking, and the birth of Louise Brown was both a medical and a media event that was celebrated as a world first.

This article will focus on Steptoe and Edwards, ‘the men who made the breakthrough’, as they were described in the *Daily Express* newspaper (11 July 1978). The press’ representation of Lesley and John Brown is to be covered separately (Dow, in preparation). Importantly, in 1978, the newspapers put Patrick Steptoe at the forefront of their reports about the pair. While much of their success was attributed to their teamwork, Steptoe, the older man who had a rather commanding manner, was assumed to be the leading figure, although the pair thought of their partnership as equal and complementary. The newspapers did not mention Jean Purdy, their clinical assistant, whom Edwards and Steptoe considered invaluable to their work (Johnson and Elder, 2015a, Johnson and Elder, 2015b).

Assisted reproductive technology continues to stimulate media interest and public debate, and Louise Brown’s birth is routinely referred back to as the origin point of any article. Of course, it is always difficult to gauge exactly what effect media has on its audience, but as Adrian Bingham (2009) reports, although newspapers had started to lose their position as the main source of news to television by the 1970s, newspaper reading was still widespread throughout British society and, he argues, reading the nationals produced in London even seemed to foster a sense of national community above and beyond local or regional identity (2009: 16). Over time, the public response to assisted reproduction has been variable, reflecting a deep-seated ambivalence, which is also true of broader attitudes to science and technology in the 20th century (Franklin, 2013, Turney, 1998). From the discovery of penicillin to the development of nuclear bombs, the 20th century brought a rapid array of new technologies that offered not only new hope for life through the eradication of disease but also the spectre of death (Edgerton and Pickstone, 2008). As Ayesha Nathoo (2009: 33–34) notes, by the 1960s, medical innovations were still celebrated in the media, but were no longer thought of as an area that needed protection from criticism. By the late 1970s, the application of investigative journalism techniques to science stories was well established. Given this, the positivity with which the British press framed IVF within the story of the birth of Louise Brown in 1978 is notable, and it underlines the point that something more was at stake than the happiness of Lesley and John Brown at the birth of their daughter.

Louise Brown was born just months before the Winter of Discontent, which marked the culmination of a series of severe industrial disputes that led to the downfall of Prime Minister Jim Callaghan, and ushered in a new era of neoliberal economic policy and increased social conservatism under his successor, Margaret Thatcher. A sense of Britain as a divided nation with profound economic troubles would have been particularly acute somewhere like Oldham, a former milling and mining town that had, at its peak, produced more spun cotton that France and Germany combined, but which now epitomized the decline of the industrial North and waning of secure employment in the latter half of the 20th century. In the dominant press narrative of the time, Steptoe and Edwards were seen to embody certain values that allowed them to be the pioneers of IVF, but they were celebrated not only for their personal success, but also with national pride. Therefore, the story of the birth of Louise Brown was, in the newspapers of the time, a story of British ingenuity and hard work. It spoke of both the moral ideology of a socially conservative historical context that celebrated ‘family values’ – which also chimed with the stance of most British newspapers – and of hope for British industry and innovation in a time of economic and political turmoil, and declining geopolitical influence.

## Materials and methods

This article takes a qualitative approach to the British press’ representation of Steptoe and Edwards in the story of the birth of Louise Brown. To illustrate the ‘success’ narrative that dominated contemporary accounts, I draw on a collection of 140 articles from across the national^3^ daily newspapers printed during 1978, as well as from the local paper, the *Oldham Evening Chronicle*, which was at the forefront of reporting the story along with the *Daily Mail,* which had made a syndication deal with the Browns, and the regional *Manchester Evening News*. The story was covered extensively across the British – and international – press throughout 1978. It broke in the *Daily Mail* and the *Oldham Evening Chronicle* on 20 April 1978, following an article in the *New York Post*. Coverage peaked in July 1978, when Louise was born, and continued until late in the year in many newspapers (although the birth was not covered in *The Sun*, the biggest-selling paper at the time, as their staff were on strike in late July and early August 1978). Notably, some of the journalists who covered the story in papers other than the *Daily Mail* and the *Oldham Evening Chronicle* were science or health correspondents, but most of the more in-depth stories were written by either generalist reporters or, in the case of the *Daily Mail*, columnists.

I have found 70 articles relating to the birth of Louise Brown printed in the *Oldham Evening Chronicle* in 1978. A group of its reporters seem to have pounced on the story, and would presumably have had the advantage of their existing knowledge of the area. Also, local people might have been more willing to talk to them than unknown journalists from London-based papers. The people of Oldham were not used to being the centre of attention in national or international news, so it is unsurprising that these local journalists put so much effort into their native scoop. Notably, Steptoe seems to have been something of a local celebrity before there was any whiff of the Louise Brown story. One article published in the *Oldham Evening Chronicle* in March 1978 – ‘Pioneer Steptoe retires in June’ – described him as ‘Oldham’s world-famous gynaecologist’. This suggests that he was a familiar face in the town, and that, in Oldham at least, he was already thought of as a pioneer of reproductive medicine before anybody knew about Lesley Brown’s pregnancy.

## ‘Baby of the century’: Steptoe and Edwards as pioneers

In her account of the first human heart transplant by Christiaan Barnard in South Africa, Nathoo notes that the regular stories of medical breakthroughs in the aftermath of the Second World War ‘continued to astound and deliver but also to unsettle’ the public (2009: 61). As she makes clear, scientists and journalists might disagree about what counts as a first, and the media will celebrate some ‘breakthroughs’ whilst criticizing or ignoring others. Yet despite this and the increasingly sceptical approach to science taken by journalists in the latter decades of the 20th century, the popularity of stories of world firsts and breakthroughs remained robust. From a journalistic perspective, having ‘pioneers’ who figured as the faces of such breakthroughs could also temper potentially dry science stories by adding drama, emotion and human interest.

A decade after Barnard’s human heart transplant, British journalists represented the birth of Louise Brown as a clear success for Steptoe and Edwards, for which they deserved acclaim and respect. Steptoe and Edwards were routinely referred to as ‘pioneers’ and ‘leaders’ in their field, and many articles rehearsed their academic credentials and years of experience. For example, the *Daily Mirror* reported, ‘The amazing birth will be a triumph for two doctors who have pioneered a method of fertilising human egg cells outside the womb’ (‘Test tube baby sensation’, Fred Austin and Peter O’Reilly, *Daily Mirror*, 21 April 1978: p.1). In this heroic narrative, their success was due to their effort, teamwork and perseverance. As the *Oldham Evening Chronicle* put it:

There were numerous set-backs and disappointments along the long, long road to success that started in the mid-1960s. But Mr Steptoe, now 65, who recently retired officially after 27 years at the hospital, refused to recognise the word failure and he pressed on with the work that has led to today’s exciting news.

The depth of feeling for his work was aptly summed up by one of his former registrars: 'He has this magnificent obsession that he will crack this problem and nothing will stop him.' (‘He’s a legend in his own lifetime’, Mike Attenborough, Janice Barker, Susan Pape and Peter O’Reilly, *Oldham Evening Chronicle*, 26 July 1978: p.13)

Some newspapers referred to public figures whose approval added moral and even political weight to the pair’s success. The *Oldham Evening Chronicle*, for example, noted that both the Vicar of Oldham, Rev. Simon Bannister, ('Steptoe experiment worth the danger', *Oldham Evening Chronicle*, 1 August 1978: p.13) and local Labour Member of Parliament Michael Meacher (‘Success welcomed’, *Oldham Evening Chronicle*, 17 August 1978: p.10) had signalled their approval of IVF, while the *Daily Mail* got as close as it could to a royal endorsement:

The Queen’s gynaecologist, Mr George Pinker, gave his full backing last night to the world’s first test-tube baby. He said of the medical team awaiting the birth of Mrs Lesley Brown’s remarkable child: ‘One has to compliment them on the fact that they have achieved this much success.’ (‘Royal surgeon hails test tube baby as a real breakthrough’, Stuart Collier, *Daily Mail*, 20 July 1978: p.3)

Steptoe and Edwards’ success was underlined by the contrast with the case of American couple Doris and John Del Zio, who had sought IVF treatment in New York in 1973 with Landrum Shettles, a well-known researcher in the field in the USA at the time (Harris, 2006, Henig, 2004). Shettles had mixed Doris and John’s gametes – notably in a test-tube rather than the more standard Petri dish – and was preparing to implant the resulting embryo into Doris’ womb. Raymond Vande Wiele, the Chief of Obstetrics and Gynaecology at the hospital, removed the embryo from the incubator in order to stop what he saw as a dangerous experiment, and was later sued by the Del Zios for damages. The case came to court in the summer of 1978, just at the same time that the world was awaiting the arrival of Louise Brown, and so a few British papers reported on it alongside her much anticipated birth. The story was portrayed as a personal tragedy for Doris Del Zio, in contrast to the happiness that Steptoe and Edwards had brought Lesley Brown.

In her close analysis of the Del Zio case, Robin Marantz Henig suggests that the contrast with Shettles conferred additional credibility to the British researchers (2004: 98, 178). Shettles was socially awkward, dismissive of authority and led a highly unconventional personal life, while Edwards and Steptoe – although ‘boffins’ – appeared respectable and benevolent in most press coverage. In fact, the British press took a largely neutral stance towards Shettles. They refrained from noting his odd appearance or abrupt manner, and only offered a negative picture of him when quoting Vande Wiele’s defence, in which he argued that Shettles lacked the skill to produce a ‘test-tube baby’ (“Doctor sued by 'test-tube baby' couple”, *The Times*, 18 July 1978: p.7). Instead, the British coverage of the Del Zio case allowed for British journalists to emphasize the fact that Steptoe and Edwards were leading the field, even compared with their colleagues in the most ‘advanced’ country in the world. As *The Guardian* put it:

Lawyers for the gynaecologist, Dr Raymond Vande Wiele, told the court that medical procedures [in the USA] were so unsophisticated five years ago that the result might have been a 'monster birth'. Comparing the techniques available at that time with those used by Mrs Leslie [*sic*] Brown’s doctors in Britain ‘is like comparing the Wright Brothers with Concorde,’ a defence lawyer told reporters. (‘US test tube baby wife tells of anguish’, Jane Rosen, *The Guardian*, 19 July 1978: p.5)

Concorde’s British–French provenance complicates the analogy somewhat, and of course Steptoe and Edwards were a long way from pulling off IVF in 1973 when Shettles attempted to achieve an in-vitro pregnancy with the Del Zios. Nonetheless, this journalist’s claim, that Edwards and Steptoe were leading the way, is very clear, as is the implication that Britain beat the USA in this scientific race.

Henig describes how, for many Americans, there was a sense of surprise and even embarrassment that they were not the pioneers of IVF. After all, in the 1950s and 1960s, America had become the leader in medical and scientific innovation, surpassing European countries including Britain in building its postwar scientific capacity (Edgerton and Pickstone, 2008: 32). Henig suggests that Howard and Georgeanna Jones, who did go on to create the first American IVF baby in 1982, must have wondered as they struggled to perfect the IVF technique, ‘How could American ingenuity fail where British and Australian perseverance had succeeded?’ (2004: 217). Edwards had worked with the Joneses at Johns Hopkins University in 1965 before he met Steptoe, but this early international collaboration was not mentioned in the British press as part of the backstory to Louise Brown’s birth, perhaps because it would have complicated the narrative of Edwards and Steptoe as lone British geniuses.

## Modesty and humanity in the press’ representation of Steptoe and Edwards

Donna Haraway describes the ‘modest witness’ of conventional modern science, in which the individual scientist is an invisible figure whose reports simply mirror reality. As she writes, ‘This self-invisibilty is the specifically modern, European, masculine, scientific form of the virtue of modesty’ (1997: 23),^4^ which ‘guarantees that the modest witness is the legitimate and authorized ventriloquist for the object world, adding nothing from mere opinions, from his biasing embodiment’ (1997: 24). Steptoe and Edwards would have been well versed in this form of modesty. While the modest witness of science scholarship is shorn of his or her human foibles and biases so as to be the mouthpiece for objective reality, modesty in news journalism is, conversely, more about being imperfect and human. In this example of reporting a potentially controversial medical technique that intervenes in the most private aspects of human life, representing the scientists and clinicians involved as modest (i.e. human) was an effective rhetorical strategy for dispelling the idea that such work is about ‘arrogant’ or ‘cold’ scientists ‘playing god’. So, while Steptoe and Edwards were routinely presented as heroic pioneers, journalists also strove to let their readers know that they were human.

While they accepted that publishing their research in academic journals was of paramount importance once Louise Brown was born, Steptoe and Edwards rejected their profession’s traditional caution towards the press, which was encapsulated in the British Medical Association’s rule against ‘indirect advertising’, and believed that they should use the media to inform the public (Edwards and Steptoe, 1980: 86; see also Seguin, 2001). Edwards and Steptoe proved mediagenic for the time. They appeared relaxed and confident on camera, willing to talk to the press and open to discussing their work. They guided the Browns in their negotiations to secure a syndication deal with Associated Newspapers, and were friendly with other journalists like the ITV film-maker Peter Williams, which suggests that they were also somewhat media savvy.

Just as the birth of Louise Brown appeared to journalists in 1978 as a winning mixture of ‘hard’ and ‘soft’ news that would appeal to a broad audience (Harris, 2006: 28), Edwards and Steptoe seemed to embody a balance of academic credentials and trustworthiness – or, to put it another way, scientific objectivity and human reliability. One way in which they established this was by describing the close relationships that Steptoe, in particular, formed with his patients. Journalists also emphasized Steptoe and Edwards’ wish to help infertile women, which sometimes shaded into overt paternalism. As the *Daily Express* reported it, ‘A former colleague of Mr Steptoe says: “We knew he would enjoy the glory, but he does care for women. He wants to solve their problems. He wants the day to come when every women can have a child.”’ (‘Steptoe’s obsession’, Harry Pugh, *Daily Express*, 26 July 1978: p.1)

Another way in which newspapers represented Steptoe and Edwards’ humanity was through their fallibility. The flipside of their hard work and dedication was the fact that they had had to overcome a series of obstacles and failures – what Edwards himself (2001) called the ‘bumpy road’ to IVF:

The happiness that shines in the faces of Lesley and John Brown as they gaze at their new-born baby is a triumphant reward for years of effort by the scientists who made the birth possible. Gynaecologist Mr Patrick Steptoe and Cambridge scientist Dr Robert Edwards have fought both man and nature for more than a decade to arrive at their historic achievement. As they tried to unravel the complex secrets of the infinitely delicate processes that governed the first faltering steps of human life, one setback after another blocked their way. ‘One is constantly being put in one’s place by nature,’ Mr Steptoe once remarked. (‘The battles – and the breakthrough’, Neville Hodgkinson, *Daily Mail*, 27 July 1978: p.4–5)

However, Steptoe and Edwards’ relationship with nature was not straightforward. Although nature could put scientists and doctors in their place, it might also be in need of help:

Mr Steptoe denies he is helping to create a ‘Brave New World’ situation in which the whole of mankind is bred and reared in test-tubes. ‘What I want to do is to help mothers whose child-producing mechanism is slightly faulty,’ he said. (‘The men who made the breakthrough’, David Thurlow, *Daily Express*, 11 July 1978: p.2)

Mr Steptoe, who lives on the moors above Oldham, does not consider himself a medical wizard or a modern Frankenstein tampering with nature. He regards his research as merely giving nature a helping nudge in the right direction. (‘The Make a Baby Doctor’, Leslie Toulson, *The Sun*, 12 July 1978: p.7)

Presumably, this second description in *The Sun* was intended to add colour to Steptoe’s personality by providing some information about his domestic arrangements. However, in British culture, moors are wild, lonely places, sometimes inhabited by lunatics, criminals and unruly characters, as in Emily Brontë’s *Wuthering Heights*, which would likely have been in people’s minds as Kate Bush’s debut single based on the story had topped the charts that January. This cultural association would have been especially salient in Oldham, given that the 1960s Moors Murders took place nearby, and that in 1978, the ‘Yorkshire Ripper’, Peter Sutcliffe, was in the midst of his misogynistic killing spree in the region. In the first of the quotes above, the *Daily Express* journalist raises the spectre of the ‘brave new world’ only to dismiss it. Such a move, notes Jon Turney, ‘reinforces the very associations it tries to deny’ (1998: 167). Similarly, with the explicit rejection of the idea of Steptoe as ‘a modern Frankenstein’ in the quote above from *The Sun*, a familiar cultural demon is invoked in order to exorcize it. Yet, raising such spectres points to a deep-seated ambivalence beneath the positive dominant narrative of the world’s first ‘test-tube baby’. As Turney (1998: 4) notes, ambivalence is a common response to scientific and technological developments, especially those that involve knowledge about how to create or modify life, with all the hopes and risks that that entails. Since the *Daily Mail* had secured the exclusive deal with the Brown family, other newspapers would have been free to paint a more negative picture of the advent of IVF or its protagonists, but here we see its main rivals indicating mild ambivalence but ultimately a positive story, and by citing Steptoe, they effectively allowed him to set the terms of the debate.

In her history of reproductive sciences in the USA, Adele Clarke describes how the endeavour has always been mired in controversy (1998: 238). As Clarke says, one of the recurring forms of illegitimacy that reproductive scientists have had to face is the association between their work and the creation of ‘brave new worlds’. What such fears point to is a rupture in the assumed relationship between human reproduction and nature. So, by interfering in ‘life itself’, reproductive sciences throw into question the ‘facts of life’ (Franklin, 1997), and they threaten what Marilyn Strathern (1992) calls the grounding function of nature – in other words, its ability to act as a fundamental and primordial reference point for all else. In the brave new world scenario, the natural order has been usurped by technology, and scientists suddenly have the power to decide who lives – and, by implication, who does not. As scholars (Squier, 1994, Van Dyck [van Dijck], 1998) have shown, these imaginaries pre-date the technological capability to create life *in vitro*, which suggests that they touch on deep-seated fears. It also means that, as Turney has pointed out, commentators have a ready arsenal of culturally salient tropes upon which to draw when expressing these anxieties.

## ‘It’s the all-British miracle’: British values in the birth of Louise Brown

While the birth of Louise Brown was reported across the world for its historic significance and its ethical implications, British journalists expressed a robust sense of national pride in this ‘all-British miracle’, as it was described in the *Manchester Evening News*. Many explicitly linked the birth’s success with its Britishness, as in this example from the *Mirror*:

The world’s first test-tube baby is due to be born in July – to a British mother. The amazing birth will be a triumph for two doctors who have pioneered a method of fertilising human egg cells outside the womb. … Now [Steptoe] has succeeded in an astonishing, all-British medical breakthrough. … Mr Steptoe’s miracle baby will be the envy of doctors around the world. (‘Test tube baby sensation’, Fred Austin and Peter O’Reilly, *Daily Mirror*, 21 April 1978: p.1)

Louise Brown’s birth was also seen as a source of local pride in Oldham. One article quoted a local councillor expressing her admiration for Steptoe: 'He and his colleagues put Oldham on the map with this miracle. Not enough praise has gone to him for what he has done, and I believe one of the greatest things ever occurred on July 25 when the first child was born.' (‘Steptoe: “No praise is great enough”’, *Oldham Evening Chronicle*, 15 August 1978: p.10) As the *Oldham Evening Chronicle* described him, Steptoe was very much the accepted incomer to Oldham, who had brought with him his expertise, upper-middle-class tastes – signified by his piano playing, white Mercedes car and sartorial style – and an altruistic desire to help – implicitly, less privileged – local women. Edwards was primarily described in terms of his academic credentials, although journalists occasionally mentioned that he had five daughters himself, thereby implying a deep-seated empathy with the desire to have children. However, the *Oldham Evening Chronicle* did not miss the fact that he was a Yorkshireman, and a profile of him included in a double-page spread about Lesley Brown’s pregnancy established his status as a northern working-class man ‘made good’ (‘5-daughter father carries eggs in rabbits’, Mike Attenborough, Janice Barker and Susan Pape, *Oldham Evening Chronicle*, 12 July 1978: p.12).

Alongside the explicit references to this birth as ‘all-British’, journalists evoked specific values in their representations of Steptoe and Edwards, which they elided with Britishness. The particular values that they referred to, and which Edwards and Steptoe thereby seemed to embody, were altruism, teamwork, industriousness and ingenuity. One editorial in the *Daily Mail* noted the hours of unpaid work that Steptoe had put into his research with Edwards: ‘There has been no official acknowledgement of the thousands of unpaid, investigatory, laborious hours he worked to perfect experimental revolutionary treatment which has brought world admiration and accolade for a British hospital’ (‘Who wants to be in the Stone Age?’ *Daily Mail*, 2 August 1978: p.7). Journalists also noted that many of the staff at Oldham worked for free over and above their usual full-time hours in order to help Steptoe and Edwards achieve the first successful IVF birth (‘And the nurses take a bow’, *Oldham Evening Chronicle*, 2 August 1978: p.12). In the late 1970s, this might well have brought to mind a comparison with the war effort in the 1940s, which many of those involved would have experienced themselves. The fact that both Steptoe and Edwards, like other men of their generation, had served in the Second World War was mentioned in some of the articles that described their backgrounds, and would surely only have added to their standing.

Others noted the simplicity of the facilities in Oldham, which the television producer Peter Williams, who made a film about Louise Brown’s birth in the summer of 1978, described to me in an interview as ‘ramshackle’ and ‘Heath Robinson’^5^ (Interview 17 March 2015), which also reflected the fact that Edwards and Steptoe had to rely on philanthropic funding for their research (Johnson and Elder, 2015a, Johnson and Elder, 2015b). One description of Steptoe and Edwards’ Oldham clinic in the *Oldham Evening Chronicle* both demystified and domesticated the modest scene of the conception of the world’s first ‘test-tube baby’:

In this tiny Royton hospital are the two secret rooms where Mr. Steptoe and Dr. Edwards carried out the delicate experiments to fertilise the eggs. … Inside, the rooms are a total disappointment for the voyeur expecting to find intricate equipment and rows of test tubes. The two small rooms contain the simplest of equipment, such as ovens, weighing scales, etc., but the work done so secretly inside them now opens up a world of hope to the one-in-ten mothers who cannot have babies because of faults in the reproductive system. (‘First test-tube baby in the world’, Peter O’Reilly, *Oldham Evening Chronicle*, 20 April 1978: p.1)

One reason that Steptoe and Edwards were able to triumph in these limited conditions was, according to journalists, their ingenuity. This is epitomized in a false and eccentric story that was reported in a handful of newspapers. The *Daily Express* described Steptoe driving to meet Edwards in Cambridge with a live rabbit in which he had implanted a human egg that was to be fertilized in Cambridge (‘The men who made the breakthrough’, David Thurlow, *Daily Express*, 11 July 1978: p.2), while the *Oldham Evening Chronicle* instead had Edwards transporting eggs that he had fertilized in his laboratory in Cambridge to Oldham using the same leporine method of incubation (‘5-daughter father carries eggs in rabbits’, *Oldham Evening Chronicle*, 12 July 1978: p.12).

Rabbits were very important in the progress of embryological research, as epitomized by Gregory Pincus’ work on IVF in rabbits in the 1930s, and Cambridge scientist LEA Rowson had flown fertilized sheep ova from the UK to South Africa, carried in a live rabbit’s fallopian tubes, in 1962 (Turney, 1998: 165). Rabbits are also colloquially associated with prodigious fertility, and in the early 20th century, they were used alongside mice and frogs in developing bioassays for pregnancy testing (see Olszynko-Gryn, 2014). The ‘rabbit test’ consequently became a euphemism for pregnancy testing, which was still in use in the 1970s. In the early stages of the research in Oldham, Edwards and Steptoe had tested whether human eggs would grow inside rabbits, and Edwards supposed this must be where this myth had come from (Edwards and Steptoe, 1980: 90; Martin Johnson, personal communication). As with all myths, the symbolism of this story is far more important than its veracity in terms of what journalists were trying to get across. The image of Steptoe and Edwards driving hundreds of miles to each other with only a rabbit for company is one that conjures up the lonely and isolated world of the maverick; the dedication, perseverance and personal cost of the research; their commitment to their partnership; the ingenious experimenter’s ability to think outside the box; and the truly Heath Robinson nature of this project.

## Discussion: time, place and character in the birth of the first ‘test-tube baby’

Three themes emerge from the way in which Edwards and Steptoe were represented in the press in 1978 – place, character and time – and they all contribute to a sense that the newspapers’ account of Louise Brown’s birth was not only about happiness for her family or personal success for Steptoe and Edwards, but also a story about Britishness winning out against the odds. Character and place are entwined in this story. Steptoe and Edwards seemed to embody the very qualities – altruism, effort, perseverance and teamwork – that had made this ‘breakthrough’ possible. Much of the often-breathless coverage of their success implied that only they could have done it. Throughout this, there was an implicit and, in the case of the Del Zio lawsuit, explicit, contrast with the USA. Compared with the New World 'upstarts' in the USA, Steptoe and Edwards’ modest research programme in Oldham appeared as the Concorde of reproductive science; their success represented British science – and, implicitly, British values – at its peak.

Rod Brookes has analysed the way in which British newspapers created a sense of national identity in the face of the bovine spongiform encephalopathy (BSE; 'mad cow' disease) crisis in the 1990s. As he shows, many journalists reinforced ‘implicit commonsense boundaries’ of ‘us’ and ‘them’ by speaking to their readers in terms of ‘we’ (1999: 250). In the BSE crisis, this was fuelled by a defensive jingoism, while in the story of the birth of Louise Brown, an apparently positive and inclusive ‘we’, tinged with triumphalism, was invoked. This story seemed to be more about national pride than xenophobia, yet as Paul Gilroy (2007) makes clear, it would be wrong to speak of British nationalism without acknowledging the role of racism, and specifically the assumption that Britain is a white nation, in British national identity, perhaps least of all when discussing the discourse of the British press. Despite the differences between the two cases and the tenor in which they were reported, Brookes’ example is helpful because it points to the normative nature of nationalistic identity-building. As he says, nationalist depictions of the nation stifle complexity and posit a naturalized political and cultural unit and a homogenized British identity. Normativity emerged in the story of the birth of Louise Brown in various ways, most obviously in the promotion of nuclear families at the heart of British society. It also emerged in the suggestion that Britain, in 1978, was a place in which hard work, ingenuity and perseverance in the face of various setbacks could lead to success.

Britain is not a country in which a unitary national identity can be easily invoked. The birth of Louise Brown concerned several parts of England: Edwards’ Cambridge, affluent and synonymous with the academic elite; the Brown family’s hometown of Bristol, popularly associated with the laidback and unpretentious West Country; and Steptoe’s adopted Oldham, a place of faded industrial glory that could also be elided with Edwards’ native Yorkshire to conjure up images of the rugged, romantic North, with its qualities of hard work, grit and traditionalism. As Brookes suggests, the invocation of British nationalism relies on the capacity to put regional and local identities to one side in favour of a shared national identity. In this case, the good news of Louise Brown’s birth provided a positive image of British success, although given Steptoe and Edwards’ associations with the North, there was also room in this narrative to claim this as a northern success story.

As noted, Britain was a divided nation at this time, not only along the lines of class and region, as seen in the rampant industrial disputes of the late 1970s, but also in terms of race – and, of course, these factors are not easily separable. Oldham was one of the places that experienced substantial immigration of workers from Commonwealth countries after the Second World War, and there has long been a strain of racialized discomfort with their presence in the town, which coincided with its industrial and economic decline. This came to a head in the Oldham riots of 2001, which were, of course, over 30 years after the birth of Louise Brown but which indicate longstanding racialized divides. Shortly after Louise’s birth, other areas of Britain experienced similar riots, including in the St Pauls area of Bristol in 1980 (which is just a mile from the Browns’ family home in Hassell Drive) and in Manchester, Leeds, London, Birmingham and Liverpool in 1981 – all fuelled by racism and economic deprivation. The peak in popularity of the far-right National Front party in the 1970s further indicates the salience of these tensions on the political landscape, although part of the reason for the party’s decline in the late 1970s seems to have been the swing back to the right of the Conservative Party under Margaret Thatcher, who was to take power in 1979. Indeed, in January 1978, Thatcher talked of British people feeling ‘rather swamped’ by immigration, and claimed, ‘We are a British nation with British characteristics. Every country can take some small minorities and in many ways they add to the richness and variety of this country. The moment the minority threatens to become a big one, people get frightened’ (*World in Action*, 27 January 1978). Given these deep divides and their political currency, it is notable that the press were able to build a picture of support for IVF that appealed to national pride in 1978. As Gilroy’s work demonstrates, however, it is important to ask what version of Britishness they evoked in the process. It would be simplistic to suggest that the birth of Louise Brown was largely celebrated because she, her parents, Steptoe and Edwards were all white, but in considering the fact that her birth and the means through which she was conceived were largely portrayed in a positive light, it is vital to bear in mind the social and political context of the time. Much of the political rhetoric of the 1970s painted Britain as a nation in decline, and for those on the right wing, this was not only a story of postindustrial downturn, but was also explicitly tied to the country's postcolonial status and the strain that they supposed migrants were placing on the economy. Given this, it is instructive to specifically consider what part this context might have played in the process of making the birth of Louise Brown appear to be more a sign of hope for the future than of science gone rogue.

Time is an important part of this story, not only in terms of taking account of the historical context, but also the temporalities that the press invoked. The pioneer trope in science and technology is a familiar one to scholars of reproductive sociology and anthropology (see Franklin, 2013, Rapp, 1999). Pioneering implies something unprecedented, a rupture between past and future possibilities, so it is important to consider what framing an event as a first, and depicting those who bring it about as pioneers, does to our understanding of it. In marking IVF in this way, there was a process of both recognition and erasure at play, in which journalists constructed a narrative of what was and was not noteworthy in the development of a medical technique that had achieved a number of firsts earlier in its history and would go on to translate into many more – and which had been claimed by other scientists previously. Framing the birth of Louise Brown as a pioneering breakthrough meant that this event would become the origin point of IVF to which all future scenarios would refer back. Pioneering evokes both nostalgia and hope; it is the ambivalent moment in which a frontier becomes visible. Nostalgia in 1978 would most likely have referred to Britain’s place at the forefront of the industrial revolution, and the ‘glories’ – as many would have viewed them then – of its recent imperial past. The hope that Louise Brown’s birth represented was not just for a cure for infertility, but also the promise of a new future of scientific discovery and technological innovation that could restore the economic fortunes of the country in which she was born. As Britain teetered on the brink of Thatcherism, this story of a home-grown scientific breakthrough showed that, despite its problems, Britain was capable of being a world leader and could still produce miracles.

## Appendix A

Bibliography

Primary Sources

Williams, P. Personal interview. London. 17 March 2015.

Newspaper articles:

‘5-daughter father carries eggs in rabbits’, Mike Attenborough, Janice Barker & Susan Pape, *Oldham Evening Chronicle*, 12 July 1978

‘And the nurses take a bow’, *Oldham Evening Chronicle*, 2 August 1978

‘First test-tube baby in the world’, Peter O’Reilly, *Oldham Evening Chronicle*, 20 April 1978

‘He’s a legend in his own lifetime’, Mike Attenborough, Janice Barker, Susan Pape and Peter O’Reilly, *Oldham Evening Chronicle*, 26 July 1978

‘Pioneer Steptoe retires in June’, *Oldham Evening Chronicle*, 7 March 1978

‘Royal surgeon hails test tube baby as a real breakthrough’, Stuart Collier, *Daily Mail*, 20 July 1978

'Steptoe experiment worth the danger', *Oldham Evening Chronicle*, 1 August 1978

‘Steptoe: “No praise is great enough”’, *Oldham Evening Chronicle*, 15 August 1978

‘Steptoe’s obsession’, Harry Pugh, *Daily Express*, 26 July 1978

‘Success welcomed’, *Oldham Evening Chronicle*, 17 July 1978

‘Test tube baby sensation’, Fred Austin and Peter O’Reilly, *Daily Mirror*, 21 April 1978

‘The battles – and the breakthrough’, Neville Hodgkinson, *Daily Mail*, 27 July 1978

‘The Make a Baby Doctor’, Leslie Toulson, *The Sun*, 12 July 1978

‘The men who made the breakthrough’, David Thurlow, *Daily Express*, 11 July 1978

‘US test tube baby wife tells of anguish’, Jane Rosen, *The Guardian*, 19 July 1978

‘Who wants to be in the Stone Age?’ *Daily Mail*, 2 August 1978

## Footnotes

1 In the newspapers’ coverage of the story, science and medicine were typically elided, and Steptoe and Edwards were described as both scientists and doctors. I have reflected this in this article by treating the press’ representation of the story of the birth of Louise Brown as a scientific *and* a medical one (and, of course, it touched on many more domains of life besides).

2 Nelkin notes that media coverage of science stories often reflects a ‘preoccupation with the existence, not the substance, of controversy’ (1987: 58).

3 These ‘national' newspapers are all, in fact, based in England and so the representations that I cite here could be described as more English than British, although one of the features of British identity is its capacity for oscillating between national, regional and local identities.

4 Gender, particularly in relation to models of masculinity, professionalism and medical authority, is clearly a very important part of this story, but unfortunately there is not space to address it explicitly in this article.

5 For those readers who are unfamiliar with this term, W. Heath Robinson was a cartoonist who drew elaborate machines (the American equivalent would be Rube Goldberg), and the term ‘Heath Robinson’ refers to an ingenious, innovative but also over-complicated and makeshift machine or device – which, importantly, always seems to succeed at its appointed task. Coincidentally, Heath Robinson also illustrated the original *Water Babies* by Charles Kingsley, an early and influential representation of ‘babies in bottles’ in English literature (see Squier, 1994: 30).
